# Animal bites and post-exposure prophylaxis in Central-West Tunisia: a 15-year surveillance data

**DOI:** 10.1186/s12879-021-06700-9

**Published:** 2021-09-27

**Authors:** Cyrine Bennasrallah, Manel Ben Fredj, Moncef Mhamdi, Meriem Kacem, Wafa Dhouib, Imen Zemni, Hela Abroug, Asma Belguith Sriha

**Affiliations:** 1 Department of Epidemiology and Preventive Medicine, University Hospital Fattouma Bourguiba, University of Monastir, Monastir, Tunisia; 2 Department of Epidemiology, Faculty of Medicine of Monastir, University of Monastir, Monastir, Tunisia; 3 Technology and Medical Imaging Research Laboratory - LTIM - LR12ES06, Monastir, Tunisia; 4 Regional Directorate of Primary Health Care of Kasserine, Kasserine, Tunisia

**Keywords:** Rabies, Animal bite, Post-exposure prophylaxis, Tunisia

## Abstract

**Background:**

Rabies is a disease that still exists in developing countries and leads to more fatalities than other zoonotic diseases. Our study aimed to describe the profile of human exposures to animals over fifteen years and to assess the post-exposure prophylaxis (PEP) practices in the governorate of Kasserine (Tunisia) on pre- and post-revolution (2011).

**Methods:**

We carried out a descriptive study using surveillance data from a region in Central-West Tunisia. All humans exposed to animals, residents in Kasserine Governorate and declared to the regional directorate of primary health care (RDPH) from January 1st, 2004 to December 31st, 2018 were included.

**Results:**

A total of 45,564 cases of human exposures to animals were reported over the fifteen-year period of the study with an annual average of 3089.2 ± 403.1. The standardized incidence rate (SIR) of human exposures to animals was 694 per year per 100,000 inhabitants (inh).

The most listed offending animal was the dog (91.3%) and the most reported type of exposure was bites (63.7%). The trend in human exposures to animals increased significantly over time. The number of exposures by vaccinated dogs decreased significantly and by unvaccinated and stray dogs increased steeply. When comparing pre-and post-revolution periods, the yearly average of animal exposures post-2011 was significantly greater than the average prior to 2011 (3200 ± 278.5 vs 2952.8 ± 483) (p < 0.001). The yearly average of animal bites post-2011 was significantly greater than the average prior to 2011 (2260.5 ± 372.1 vs 1609.8 ± 217.9) (p < 0.001). The average number of vaccine doses per animal exposure was 2.4. Concerning PEP protocols, protocol A (2 and 3 doses) was indicated in 79% of animal exposures cases. From 2004 to 2018, a downward trend was noted for protocol A (r = − 0.29, p < 0.001) and an upward trend for protocol B (3 and 5 doses) (r = 0.687, p < 0.001). During our study period, 5 fatal cases of human rabies were declared.

**Conclusion:**

Rabies remains a major public health problem in Tunisia. The political dynamics had an impact on the health care system and rabies control. Preventive measures should be applied adequately to decrease the burden of this disease.

## Background

Rabies is a neglected zoonotic disease caused by an RNA virus of the genus *Lyssavirus* of the *Rhabdoviridae* family [1]*.* It is an infectious viral disease that affects the central nervous system of mammals and is fatal once symptoms develop [2]. Rabies is a disease of antiquity, still existing, and leading to more fatalities than any other zoonotic diseases [3, 4]. Bites and scratches are the two main modes of rabies transmission from animal to human but other modes are possible such as licking [5]. Almost 59 000 human deaths annually are attributable to rabies, mostly among underserved populations in Africa and Asia [6]. Africa is responsible alone for 43% of human rabies death [7]. Over 95% of human deaths result from virus transmission through rabid dog bites [8]. Fortunately, it is a preventable disease through pets vaccination, adequate management of bite wounds, timely post-exposure prophylaxis (PEP) and public health interventions to control stray animals [9]. However, the complete eradication of rabies is a public health challenge. This is due to the nature of causal agent living in a wide variety of hosts on the one hand and to the status of the disease as neglected in the other hand [10]. Moreover, the lack of information on the endemicity and burden of the disease contributed in part to hinder the elimination of this fatal disease [6].

Rabies is endemic in North Africa and thus, the development of an elimination program through dog vaccination and PEP provided to people exposed to suspected rabid animal should be encouraged [2]. In Tunisia, rabies is a notifiable disease with a national surveillance system in place [2]. The National Rabies Control Program (NRCP) was implemented in 1982, by a collaboration of the Ministry of Interior, the Ministry of Agriculture and the Ministry of Health. This program focused on implementing dog mass vaccination, providing PEP to people who was exposed to suspected rabid dogs and maintaining epidemiological surveillance activities in order to detect all suspect cases [11].

Dog vaccination campaigns have been carried out on a yearly basis since 1993 [2]. It was free of charge for dogs’ owners. Similarly, PEP was provided for free in the public sector and was consistently available in accordance with a Tunisian law published in March 2003 [11]. PEP consists of wound washing, vaccine, and in some cases rabies immunoglobulin (RIG). Following the national strategy, a significant improvement in rabies health status in Tunisia was noted with a decrease in animals and humans’ cases [2].

The Tunisian revolution took place in January 2011 and many democratic gains were achieved. However, these political dynamics had impacted the performance of the health care system in a negative way which is common at moments of crisis or instability [3]. A significant upsurge in cases of rabies in dogs and humans was reported in many governorates and the epidemiological situation became alarming [12]. Instability and insecurity caused by the manifestations led to a decline in dog vaccination coverage and adverse consequences on the disease surveillance [2]. Veterinarians were unable to collect samples and a variation in reporting cases was noted [12]. Indeed, A drastic decrease in the number of submitted samples was observed ( 282 samples analyzed in 2011 compared to 415 in 2009) [13].

A mismanagement of stray dogs has also been noted since 2011. Stray dog proliferation and the inaccessibility to such population may have influenced the vaccination coverage and limit the result of the control program. The NRCP strategy to control stray dogs were based on slaughter aiming to reduce the risk of infection to humans. This controversial practice was debated after the revolution, between the government and the civil society resulting in its suspension and growth in stray dog population. Controlling the stray dog population is a municipality responsibility. However, the lack of resources and personnel may be a barrier to appropriately facing their responsibilities [14]. The overall coverage vaccination varies from a region to another. Kasserine governorate is one of the regions with a low vaccination coverage in Tunisia and where a reservoir for the disease is maintained [15]. Vaccination coverage was 59% in Kasserine with an only 48% coverage in the urban area compared to 70% and 71% in Mannouba and Siliana governorates respectively [16].

Few published studies are available about human exposures to animals before and after 2011 making it essential to measure the impact of the Tunisian revolution on rabies status.

Our study aimed to describe the epidemiological profile of human exposures to animals over fifteen years and to assess the PEP practices in the governorate of Kasserine (Tunisia) pre- and post-revolution.

## Methods

### Study design

We carried out descriptive study using a surveillance data over 15 years, from 2004 to 2018.

### Setting

The governorate of Kasserine is located in the Central-West of Tunisia. It covers an area of 8260 km^2^ and is divided into 13 delegations (El Ayoun, Ezzouhour, Ferida, Foussana, Haidra, Hassi el Ferid, Jedelienne, Kasserine north, Kasserine south, Majel el Abbes, Sbeitla, Sbiba, Tala) [17]. It is predominantly an urban area estimated at 439,243 inhabitants (inh) in 2014; children under 15 accounted for 27% of the general population [17].

### Participants

All suspected exposures, residents in Kasserine Governorate and declared to the Regional Directorate of Primary Healthcare (RDPH) from January 1st, 2004 to December 31st, 2018 were included in our study. Cases living in another governorate were not included.

### Operational definition and variables

An exposure was defined as suspect if the animal bit a human without any reason, if the animal was wild or unvaccinated, or if the animal escaped or died after the bite [14].

Stray dogs were defined as ownerless dogs with no health care, having to forage for their own food [18].

The variables of interest were: Demographic variables (age, sex), culprit animals and their vaccination history, types of exposures (bites, scratches, licking, others (including the contamination of open wounds or mucous membranes, abrasions) [19]), sites of exposures, reported cases of human rabies, annual number of vaccine doses distributed, as well as annual post-exposure prophylaxis (PEP) protocol adopted (protocol A and B). The number of vaccine doses administered per exposure was calculated by the proportion of vaccine doses distributed per year over the total of number of exposures per year.

Protocol A and B are the PEP protocols adopted in Tunisia for unvaccinated victims. They depend on the immunization status of the offending animal. All animals are considered as possibly rabid due to the endemic nature of rabies in this area. Protocol A is administered if there is a contact with a known animal, in apparent good health and under observation. And protocol B is adopted if the culprit animal is unknown, lost sight of, dead or was confirmed being rabid.

Protocol A and B are divided into (A1/A2) and (B1/B2) depending on the severity of the injury.

**Fig. 1:**
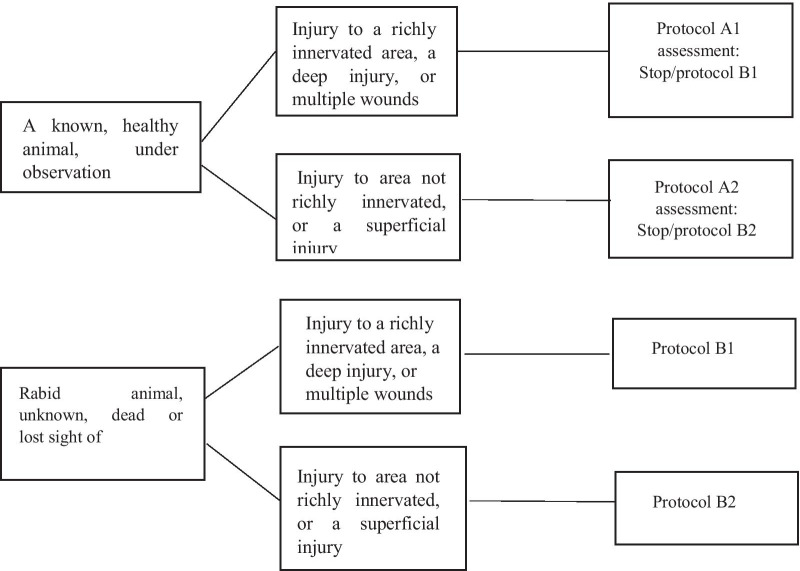
Rabies post-exposure prophylaxis (PEP) flowchart

Protocol A1 and B1 are administered intramuscularly when the injury is deep, consists of multiple wounds, or is located in a richly innervated area such as the head, neck and/or extremities (Fig. 1).

Protocol A1 consists of 2 vaccine doses on days 0 and 3 and doses could be pursued or stopped on the 7^th^ day according to the veterinary observation of the responsible animal. Otherwise, protocol B1 consists in 5 doses on days 0, 3, 7, 14 and 28. Furthermore, rabies immunoglobulin (RIG) is injected on day 0 in both protocols.

Protocol A2 and B2 are administered intramuscularly when the injury is superficial or not in a richly innervated area. For protocol A2, three vaccine doses are indicated; two doses are administered upon the first contact with the victim and the third dose in a week, depending on the veterinary observation of the culprit animal. As for protocol B2, a supplementary dose is recommended on day 21.

### Data sources/measurement

Data was collected from the RDPH of Kasserine. There are 6 regional rabies centers in Kasserine with 3 human centers and 3 veterinary centers [11]. The national referral laboratory for human and animal rabies diagnosis in Tunisia is the Pasteur Institute in the capital city Tunis. The RDPH defines confirmed rabies cases as those with paralytic or furious symptoms of rabies and have been confirmed by the detection of rabies viral antigens by direct fluorescent antibody (DFA) or by polymerase chain reaction (PCR) assay. This test finds the genetic material (RNA) of the rabies virus protein. PCR can be done on saliva, cerebrospinal fluid, or tissue.

### Data analysis

The data was analyzed statistically using SPSS (Statistical Package for Social Sciences), version 21 software. Categorical variables were reported as count and percentages. Quantitative variables were expressed in terms of means and standard deviation.

The standardized incidence ratio (SIR) is calculated as the ratio of the observed number of cases to the expected number of cases. The observed number of cases refers to the number of cases in the study population of interest. The expected number of cases is computed using age-specific rates from a reference population, weighted according to the age structure of the study population [20].

The chi-squared test was used to assess the association of categorical variables. The means of annual animal bites before and after 2011 were compared using an independent sample t test.

Linear regression was used to estimate trends.

A p-value less than 0.05 was considered significant at 95% confidence level.

### Ethical considerations

Ethics Committee of the faculty of medicine of Monastir approved the protocol of this study. To maintain the principle of confidentiality, the data used were anonymized.

## Results

### Characteristics and incidence of human exposures to animals

A total of 45,563 cases of human exposures to animals were reported over the fifteen-year period of the study with an annual average of 3089.22 ± 403.10 cases.

By gender, significantly more males were victims to animal exposures than females. By age classes, the majority of victims were aged between 5 and 14 years old. Crude incidence rate (CIR) and standardized incidence rate (SIR) of human exposures to animals were almost the same (Table 1). As for bites, the CIR was 441 per 100,000 inh.

**Table 1:** Reported cases of exposures to animals according to the age and gender

	Exposures	Percentage (%)	p	CIR/ year /100,000 inhabitants	SIR/ year /100,000 inhabitants
Gender					
Males	26,145	57.4	< 0.001	209	–
Females	19,418	42.6		82	–
Age					
1–4	2467	7.2	< 0.001	375	–
5–14	12,637	27.7		1063	–
15–29	7838	17.2		421	–
≥ 30 years	21,773	47.7		760	–
Total	45,564	100		693	694

*CIR* crude incidence rate, *SIR* standardized incidence rate

Table 2 illustrates the characteristics of animal exposures before and after the revolution. The majority of human exposures to animals (91.88%) were caused by dogs (p < 0.001). The injury site was head and face in 5.3% of cases, while injury to the extremities was reported in a quarter of cases (26%). Other lesions were seen in the majority of cases. Regarding the type of exposure, bites were reported by two thirds of victims (n = 29,062, 63.7%) followed by scratches (n = 8214, 21.2%) and licking (n = 1466, 3.7%) (p < 0.001).

**Table 2:** Characteristics of animal exposures before and after the Tunisian revolution

Characteristics	Total (N = 45,564)		Before 2011 (N = 20,104)		After 2011 (N = 25,460)		p-value Before vs After 2011
	N	%	N	%	N	%	
Animal type							
Dogs	41,830	91.88	18,761	93.32	23,069	90.75	0.001
*VD*	16,017	35.18	9392	46.72	6625	26.06	0.001
*UVD*	19,392	42.60	7227	35.95	12,165	47.86	0.001
*SD*	6421	14.10	2142	10.65	4279	16.83	0.001
Cats	2194	4.82	699	3.48	1495	5.90	0.001
Cattles	1017	2.25	472	2.35	545	2.14	0.001
Others	480	1.05	172	0.85	308	1.21	0.001
p-value*	0.001		0.001		0.001		
Injury site							
Head and face	1996	4.3	948	4.71	1048	4.1	0.001
Extremities	11,833	26	4479	22.2	7354	28.8	0.001
Others	31,734	69.7	14,677	73	17,057	66.99	0.001
p-value**	0.001		0.001		0.001		
Types of exposure							
Bites	29,062	63.7	11,148	55.45	17,914	70.39	0.001
Licking	1466	3.78	663	3.29	803	3.1	0.001
Scratches	8214	21.2	3903	19.04	4311	16.9	0.001
Others	6811	17.5	4390	21.8	2421	9.5	0.001
p-value***	0.001		0.001		0.001		

*VD* Vaccinated dogs, *UVD* Unvaccinated dogs, *SD* Stray dogs

p-value*: comparison between animal types; p-value**: comparison between injury sites; p-value***: comparison between types of exposure

**Fig. 2:**
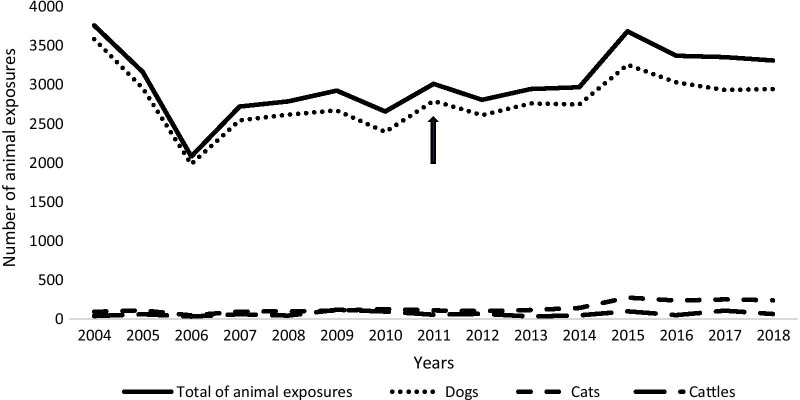
Reported exposure cases according to the animal type

Overall, animal exposures incidence trend increased significantly over time (r = 0.263, p < 0.001). As for dog bites, a positive trend was also noted (r = 0.850, p < 0.001) (Fig. 2).

When comparing pre- and post-revolution periods, we have found that the yearly average of animal exposures post-2011 was 3200 ± 278.5 which is significantly greater than the average of 2952.8 ± 483 prior to 2011 (p < 0.001).

Similarly, the yearly average of bites post-2011 was significantly greater than the average in pre-2011 period (2260.5 ± 372 vs 1609.8 ± 217, p < 0.001).

### Dog vaccination and post-exposure prophylaxis

During the study period, 35% of the total of offending dogs were reported as vaccinated.

**Fig. 3:**
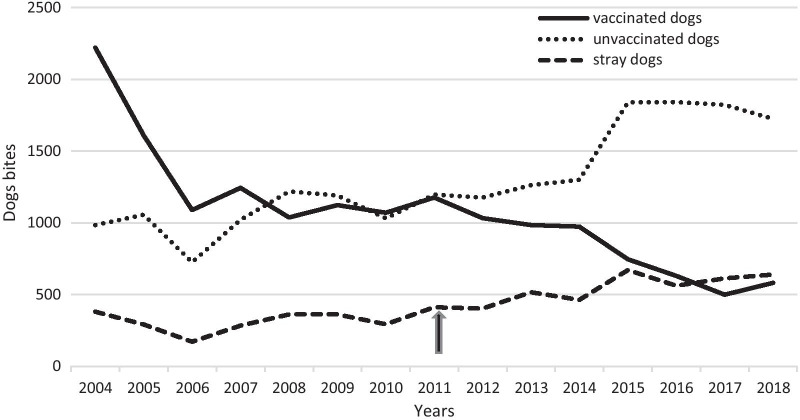
Dog bites number per year according to the immunization status

In addition, a remarkable decrease in the number of exposures by vaccinated dogs was observed (r = − 0.847) (p < 0.001). However, a steep increase in bites by unvaccinated and stray dogs was detected with (r = 0.874) and (r = 0.860) (p < 0.001) respectively (Fig. 3).

Dog bite rates were compared before and after 2011 according to their immunization status and also reported in Table 2.

Post-2011, the annual average of vaccinated dog bites was 818.68 ± 229 cases per year, significantly less than the average of 1403.68 ± 229 per year prior to 2011. Moreover, after the revolution yearly averages of unvaccinated and stray dog bites were respectively 1537.03 ± 290 and 540.55 ± 97 cases per year, which is significantly higher than the respective annual averages before the revolution 1041.26 ± 137 and 314.50 ± 62 cases per year.

The total number of vaccine doses distributed by the RDPH are summarized in Table 3 with an average number of vaccine doses at 2.4 per animal exposure. The number of vaccine doses per animal exposure were almost equal pre- and post-revolution, with an average of 2.3 doses per exposure between 2004 and 2010 and 2.4 doses per exposure between 2011 and 2018. During our study period, protocol A was indicated in 79% of cases of human exposures to animals.

**Table 3:** Total of vaccine doses distributed and post-exposure prophylaxis adopted by protocols

Years	Cases	Vaccine		Post-exposure prophylaxies Protocol (PEP)	
	Exposures*	Total doses Distributed	Doses per exposure	Protocol A	Protocol B
2004	3759	8299	2.2	3262	497
2005	3166	7383	2.3	2731	435
2006	2084	4578	2.2	1853	231
2007	2723	6422	2.3	2357	366
2008	2788	7939	2.8	2296	492
2009	2925	7185	2.4	2319	606
2010	2659	6159	2.3	2162	497
2011	3012	7314	2.4	2429	583
2012	2808	7000	2.5	2233	575
2013	2946	7408	2.5	2205	741
2014	2969	7160	2.4	2237	732
2015	3686	9056	2.4	2348	1338
2016	3372	8507	2.3	2543	830
2017	3355	8531	2.5	2519	836
2018	3311	8527	2.5	2502	809
Total	45,564	111,468	N/A	35,996	9568
Annual average	3037.6	7431.2	2.4	23997	637.9

* Includes bites, scratches, licking and others

From 2004 to 2018, a downward trend was noted for protocol A (r = − 0.29, p < 0.001) and an upward trend for protocol B (r = 0.687, p < 0.001) (Table 3).

### Human rabies cases

During our study period, 5 cases of human rabies were declared to RDPH. All of the 5 cases resulted in death and were associated with dog bites. No dog had a history of rabies vaccination. A failure of PEP was noted for two victims with an inappropriate sutured wound in one case and an incomplete PEP protocol in the other case (Table 4).

**Table 4:** Reported human rabies cases from 2004 to 2018 in Kasserine, Tunisia

Year	2004	2005	2010	2015	2017
Cases	1	1	1	1	1
Age	21	18	18	12	33
Animal	Stray dog	Unvaccinated dog	Stray dog	Unvaccinated dog	Stray dog
PEP	No	No	No	Yes (Complete)	Yes (Incomplete)

*PEP* Post-exposure prophylaxis

## Discussion

To the best of our knowledge, this is one of the few studies to describe the epidemiological profile of human rabies and animal bites and to assess the PEP practices in a region of a developing country (Tunisia). It might provide a reliable epidemiology basis for further rabies control and prevention.

Our results showed that the overall incidence of animal bites was 441 per 100,000 inh in Kasserine. This incidence was much higher than other studies conducted in some endemic countries for rabies in the MENA region [3, 21, 22]. Our investigation showed that most exposures were attributed to dogs, suggesting that control efforts should focus on them.

Males were more frequently injured by animals than females. This was similar to results from other studies in Iran, Oman and Ethiopia [21–23]. Children aged between 4 and 15 years had the highest exposure to suspected rabid animals in line with the WHO reports [24]. This could be attributed to the greater engagement in outdoor activities among this age group and might have thereafter a negative impact on the national economy [23]. A Nigerian study noted that a higher level of education of school age children was correlated with a lower risk of rabies [25]. Thus education of children on the dangers of dog bites, could play a role in reducing the burden of rabies [26].

Our data showed, a positive trend in the number of animal exposures from 2004 to 2018. Annual averages of animal exposures and bites were greater in post-2011 than in pre-2011. However, from Figs. 2 and 3, the most notable change occurred in 2014–2015. It could be the consequence of the political and social instability since 2011 reaching its peak in 2014–2015. The Tunisian revolution and its impact on the health system are involved in this alarming trend. This is comparable with the situation in Lebanon since the Syrian crisis [3].

Our data showed a decrease in bites from vaccinated dogs and an increase in those from unvaccinated ones which can reflect a decline in vaccination level. This could be explained by a decrease in the canine mass control and rabies monitoring in recent years.

Despite the free of charge vaccination for dog owners and the annual dog immunization campaigns, only a 59% coverage was obtained in 2011 in Kasserine Governorate [16]. This is far from the 70% recommended by the WHO for effective rabies control [27]. In fact the high turnover in dog population, could result in decreasing the herd immunity effect between vaccination intervals and so not reaching the rabies control rate [28]. Interventions that reduce the turnover like castration or contraception and enhancing life expectancy might slowdown the decline in dog vaccination coverage and thus, contribute to its improvement [29].

Other factors led to the low level of canine immunization in developing countries including Tunisia such as, the lack of appropriate public health laws and the poor implementation of existing legislation on animal ownership and immunization [26].

Interestingly, mass vaccination should target domestic dogs in order to have the highest impact in reducing rabies infection, explained by the fact that domestic dogs represent the main virus cycle maintenance in Africa [30, 31].

During our study period, the annual average of vaccine doses per bite was 2.4. This average was less than the 3 and 5 doses recommended by protocol B for unvaccinated and stray dogs which was the predominant status of culprit animal according to our database. In fact, culprit animals were mostly stray and unvaccinated dogs requiring protocol B1 and B2 with 3 and 5 doses recommended for each one respectively. However, the protocol A was the most adopted. This showed an inadequate prescription of protocols which could be explained by the complexity of the Tunisian PEP regimen causing ambiguity among health workers. Therefore, to make it easier and for a better compliance, protocols A and B may be replaced by a unique four or five-dose IM regimen as recommended by the WHO [32].

In addition, the lower averages of vaccination doses than recommended, could be explained by the lack of adherence to PEP by victims, because of the poor awareness of rabies severity or inadequate access to basic health care services in some rural areas [33].

Low annual averages of vaccine doses could also be attributed in part to the interruption of PEP because animals were healthy and alive after the observation period.

Poor management of wounds and poor adherence to PEP resulted in two fatal human rabies cases after 2011. In 2015, a 12-year-old boy died despite post- exposure vaccination from an unvaccinated dog bite. The RDPH investigation revealed a history of suturing the wound and a poor management of the injury as the cause of death. However, suturing of mammalian bites remains controversial [34]. A recent report from the WHO showed that wounds should not be sutured. However, if closure is necessary, suturing should be done after infiltration of the wound with RIG and should be loose and not interfere with free bleeding and drainage. Therefore, the management of dog bites need to be improved by health services in order to prevent tragic deaths from rabies, such as those reported in our study [26]. In 2017, another fatal case was notified due to a loss of follow up and an unfinished PEP protocol. Similarly, a recent study in Tanzania reported that rabies victims may not return to complete the full course of prophylactic vaccine [35].

Finally, this study highlighted the importance of the “one health concept” to achieve the goal of the WHO and the World Organization for Animal Health (OIE) to eliminate dog-mediated rabies deaths by 2030. This concept looks at health in the context of human, animal and their shared environment. It requires collaborative efforts including without limitations physicians, veterinarians, the media, authorities and community’s involvement. Rabies-endemic countries such Tunisia needs to collaborate together and share their experiences in order to eliminate the disease. Special rabies control committees under the “one health prospective “should be implemented. To achieve 2030 goals, enforcing legislation, allocating resources and the assessment of the national rabies control programs weaknesses by governments are key solutions [36].

Interestingly, in the context of our rabies endemic country intradermal vaccination is an acceptable alternative to standard intramuscular vaccination as it is safe, immunogenic and dose and cost sparing [37].

Our study provided useful information on the epidemiology of rabies in an endemic region and contributed to assessing the PEP practices between pre- and post-2011 periods. However, our study had some limitations. Firstly, the under reporting of notified cases because of rabies passive surveillance system may under-estimate the real incidence rate of animal exposures to humans. Secondly the lack of consistency in the record keeping during the covered years, and potential modifications of administrative data collection led to database deficiencies especially with rabies risk assessment after observation. Another limitation of our study was the limited geographical coverage by incorporating data of one governorate which may not reflect the situation in the other regions of our country. Indeed, additional nation-wide studies are needed to better understand the characteristics of rabies in our country.

## Conclusions

Rabies remains a major public health problem in Tunisia. Unfortunately, the promising results of the NCRP in the beginning of its implementation, have not been maintained and the Tunisian revolution in January 2011 had impacted the health care system and rabies control. It is crucial to apply and develop the appropriate preventive measures such as enhancing mass dog vaccination, controlling stray dog populations and training medical personnel to provide adequate PEP. In addition, public education on rabies prevention should be enforced through media and by organizing awareness days in order to decrease rabies risks. Many developing countries should be strict in applying recommendations on rabies control to achieve the WHO goal of zero human deaths from dog-mediated rabies deaths by 2030.

## Data Availability

The datasets generated and/or analyzed during the current study are not publicly available due to reasons of patient confidentiality but are available from the corresponding author on reasonable request.

## Abbreviations

WHO: World Health Organization
OIE: World Organization for Animal Health
PEP: Post-exposure Prophylaxis
CIR: Crude incidence rate
SIR: Standardized incidence rate
VD: Vaccinated dogs
UVD: Unvaccinated dogs
SD: Stray dogs
IM: Intramuscular
